# Structural and biochemical characterization of the exopolysaccharide deacetylase Agd3 required for *Aspergillus fumigatus* biofilm formation

**DOI:** 10.1038/s41467-020-16144-5

**Published:** 2020-05-15

**Authors:** Natalie C. Bamford, François Le Mauff, Jaime C. Van Loon, Hanna Ostapska, Brendan D. Snarr, Yongzhen Zhang, Elena N. Kitova, John S. Klassen, Jeroen D. C. Codée, Donald C. Sheppard, P. Lynne Howell

**Affiliations:** 1 Program in Molecular Medicine, The Hospital for Sick Children, Toronto, ON M5G 1X8 Canada; 2 Department of Biochemistry, Faculty of Medicine, University of Toronto, Toronto, ON M5S 1A8 Canada; 3 Department of Microbiology and Immunology, Faculty of Medicine, McGill University, Montreal, QC H3A 2B4 Canada; 4 Infectious Disease and Immunity in Global Health, Research Institute of McGill University Health Center, Montreal, QC H4A 3J1 Canada; 5 McGill Interdisciplinary Initiative in Infection and Immunity, Montreal, QC H3A 1Y2 Canada; 6 Leiden Institute of Chemistry, Leiden University, 2300RA Leiden, The Netherlands; 7 Alberta Glycomics Centre and Department of Chemistry, University of Alberta, Edmonton, AB T6G 2G2 Canada; 8 Present Address: Division of Molecular Microbiology, School of Life Sciences, University of Dundee, DD1 5EH Dundee, UK

**Keywords:** Glycobiology, Biofilms, Fungi, X-ray crystallography

## Abstract

The exopolysaccharide galactosaminogalactan (GAG) is an important virulence factor of the fungal pathogen *Aspergillus fumigatus*. Deletion of a gene encoding a putative deacetylase, Agd3, leads to defects in GAG deacetylation, biofilm formation, and virulence. Here, we show that Agd3 deacetylates GAG in a metal-dependent manner, and is the founding member of carbohydrate esterase family CE18. The active site is formed by four catalytic motifs that are essential for activity. The structure of Agd3 includes an elongated substrate-binding cleft formed by a carbohydrate binding module (CBM) that is the founding member of CBM family 87. Agd3 homologues are encoded in previously unidentified putative bacterial exopolysaccharide biosynthetic operons and in other fungal genomes.

## Abstract (web summary)

The exopolysaccharide galactosaminogalactan (GAG) is an important virulence factor of the fungal pathogen *Aspergillus fumigatus*. Here, the authors study an *A. fumigatus* enzyme that deacetylates GAG in a metal-dependent manner and constitutes a founding member of a new carbohydrate esterase family.

## Introduction

**Fig. 1:**
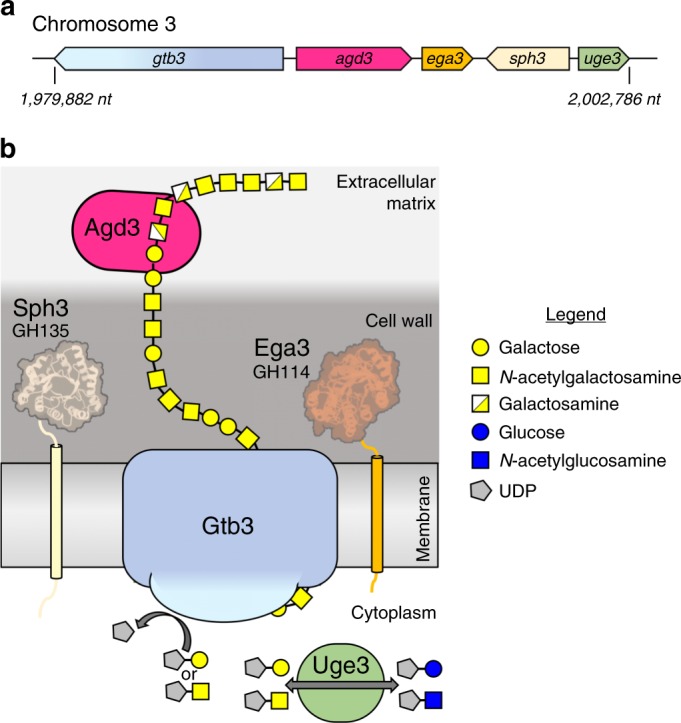
Current model of GAG biosynthesis in *A. fumigatus*. **a** Schematic of the five co-regulated genes of the GAG cluster found on chromosome 3. The location on the chromosome is is denoted by nucleotide (*nt*) number. **b** Cartoon representation of the GAG biosynthesis system based on bioinformatics and previous studies including the two crystal structures of the glycoside hydrolase domains of Sph3 (PDB 5C5G) and Ega3 (PDB 6OJ1). The synthesized polymer is hypothetical in sequence as the GAG polymer is heterogeneous.

*Aspergillus fumigatus* is a ubiquitous, biofilm-forming, filamentous fungus that causes invasive infections in immunocompromised patients^1^. Even with currently available antifungal agents, the mortality of invasive aspergillosis remains over 50%, highlighting the need for new therapies and better understanding of the underlying virulence factors governing *A. fumigatus* pathogenesis^2^. During infection *A. fumigatus* transitions to a biofilm mode of growth in which fungal hyphae are encapsulated in a self-produced matrix^3^. Galactosaminogalactan (GAG), an α-1,4-linked linear exopolysaccharide of galactose (Gal) and *N*-acetylgalactosamine (GalNAc), is essential for *A. fumigatus* biofilm formation and a key virulence factor^4^. The synthesis of GAG is dependent on a cluster of genes encoding five carbohydrate-active enzymes (Fig. 1)^5,6^. A model of GAG biosynthesis and modification has been proposed (Fig. 1) and includes the production of activated monosaccharide building blocks by the epimerase Uge3^5,7^ followed by synthesis and export by the predicted integral membrane glycosyltransferase Gtb3^5^. The GAG cluster also encodes two glycoside hydrolases: an endo-α-1,4-*N*-acetylgalactosaminidase, Sph3^6,8^; and an endo-α-1,4-galactosaminidase, Ega3^9^. Lastly, GAG is partially deacetylated by the secreted protein Agd3 and deletion of *agd3* abolishes this modification^5^. Although, the ∆*agd3* mutant produces normal amounts of GAG, this strain has markedly impaired biofilm formation and lacks the cell-wall decoration associated with GAG production^5^. The ∆*agd3* mutant also exhibits significantly lower virulence in a murine model of *A. fumigatus* infection compared with the WT strain, supporting Agd3 as a virulence factor^5^. GAG synthetic gene clusters containing Agd3 homologs have been identified in numerous fungal species including plant and animal pathogens^5^.

Deacetylation is important in exopolysaccharide processing and production in multiple bacterial biofilm systems including *Staphylococcus aureus*, *Staphylococcus epidermidis*, *Escherichia coli*, *Bordetella bronchiseptica*, *Listeria monocytogenes*, and *Pseudomonas aeruginosa*^10–17^. De-*N*-acetylation of biofilm exopolysaccharide has been linked to cell aggregation, surface attachment, exopolysaccharide secretion, and biofilm maturation depending on the organism^13–17^. In each of these systems, de-*N*-acetylation is mediated by a protein that belongs to carbohydrate esterase (CE) family 4. Agd3 has low sequence homology to the CE4 family and does not belong to any of the currently defined Carbohydrate Active enZyme database (CAZy) CE families.

Herein, we show that *A. fumigatus* Agd3 has a unique three-domain architecture and that the enzyme specifically deacetylates α-1,4-GalNAc oligosaccharides in a metal-dependent manner. Agd3 has a novel carbohydrate-binding module (CBM) that extends the substrate binding groove, increases activity on soluble GAG, and influences the location of deacetylation within oligosaccharides. Our structural and functional characterization of the CE-domain and N-terminal domain of Agd3 presented herein reveal that these domains are the founding members of CE18 and CBM87 families, respectively. Phylogenetic analysis found distant homologs in the bacterial kingdom. Although not all *agd3*-like genes are located in operons, those that were co-localize with other putative carbohydrate-active enzymes, resided in uncharacterized exopolysaccharide biosynthetic clusters. These findings suggest deacetylation of a carbohydrate polymer by an Agd3 homolog is a more prevalent occurrence than previously appreciated and will aid in further genome annotation.

## Results

### Agd3 has low homology to CE4 family members

Bioinformatics has suggested that Agd3 is a secreted 806 amino acid multidomain protein^5^. The N-terminus of the protein, residues 38–129, contains a serine-rich region that is predicted to be structurally disordered by Phyre^2^^18^. When summited to NetOGlyc 4.0 server^19^, 69 O-glycosylation sites were identified between residues 26 and 141. Although NetOGlyc is designed for predicting sites of O-glycosylation on mammalian peptides it has been shown to accurately predict areas of hyper-O-glycosylation in fungal proteins^19,20^.

Agd3 was also predicted to contain a domain, residues 509–726, with structural similarity to members of the carbohydrate esterase family 4 (CE4)^5^. The CE4 family contains exopolysaccharide deacetylases that adopt a (β/α)_7_-fold. As previously noted, Agd3 has very low sequence similarity to the CE4 family and has a predicted CE domain encompassing at least 370 residues. This is in contrast to known CE4 family members where the CE domain is typically under 300 residues^5,21^. The amino acid sequence of Agd3 is too divergent to be a member of the CE4 family, thus direct evidence of carbohydrate deacetylation would allow classification of Agd3 as the founding member of a CE family. With the exception of the predicted domain, with structural similarity to CE4’s, Agd3 had little predicted similarity to other biochemically or structurally characterized proteins. An 88-residue stretch, residues 147–234, was predicted to be structurally similar to amido-transferases but this region did not contain the catalytic residues required for these enzymes^5^. The function of this domain and the C-terminal β-rich domain is unknown.

### Agd3 has a compact three-domain architecture

**Fig. 2:**
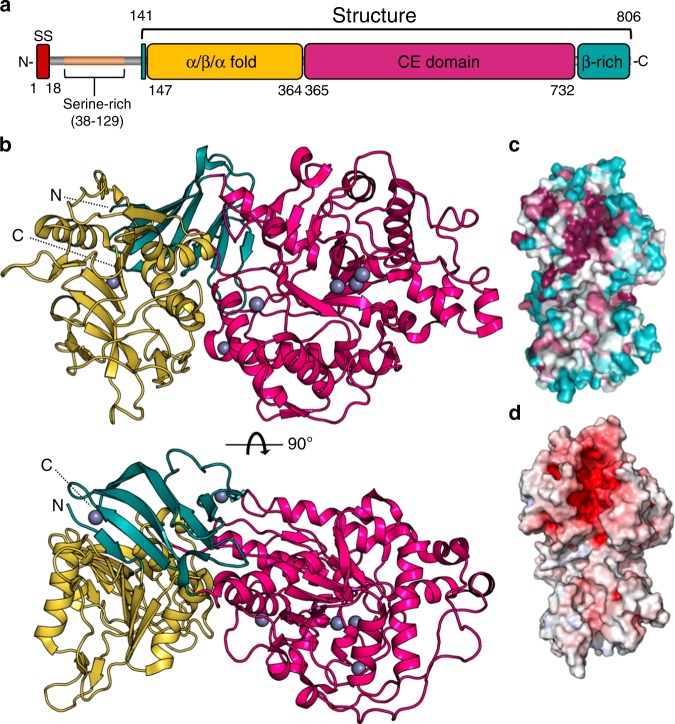
Agd3 is a multi-domain protein with a conserved electronegative cleft. **a** Linear representation of Agd3 domains based on the structure. **b** Agd3 in cartoon representation displaying the three-domain arrangement: the N-terminal α/β/α-fold in yellow; the catalytic CE-domain in pink; and the C-terminal β-rich domain in teal. Zinc ions are depicted as gray spheres. **c** Surface representation of amino acid conservation colored from variable (teal) to conserved (fuchsia) as calculated by Consurf^85^. **d** Electrostatics surface representation shows an electronegative cleft. Electrostatics were calculated by APBS in PyMol (v. 2.0.7) and visualized in blue to red (+10 kT/e to −10 kT/e)^86^.

To gain insights into the function of Agd3, structural studies of the predicted globular protein, residues 141–806, were pursued. Agd3 crystallized in the hexagonal space group *P* 6_1_ 2 2. Diffraction data were anisotropic with diffraction limits of 2.6 Å in one dimension and 3 Å in the other two dimensions. The structure was solved by Zn single-wavelength anomalous dispersion and refined to *R*_work_ and *R*_free_ of 19% and 24%, respectively (Supplementary Table 1). Agd3 has a unique compact three-domain structure (Fig. 2a,b). The N-terminal domain, comprising residues 141–364, adopts a mixed α/β/α-fold (α/β/α-domain) with a seven-stranded β-sheet at its center. The central domain, residues 365–733, contains the CE4 homology region and has a distorted (β/α)_7_-fold (CE-domain). The C-terminal domain spanning residues 733–806 forms six β-strands in a seven-stranded β-sandwich. The seventh strand is contributed by the N-terminal region residues 146–149, residues prior to the β/α/β-domain. The N- and C-terminal domains increase the stability of the CE-domain, as constructs lacking one or both of these accessory domains expressed poorly and could not be purified to homogeneity from either *E. coli* or *Pichia pastoris*. Mapping surface conservation reveals a highly conserved electronegative cleft in the CE-domain (Fig. 2c,d).

### Agd3 contains a unique CE-domain

**Fig. 3:**
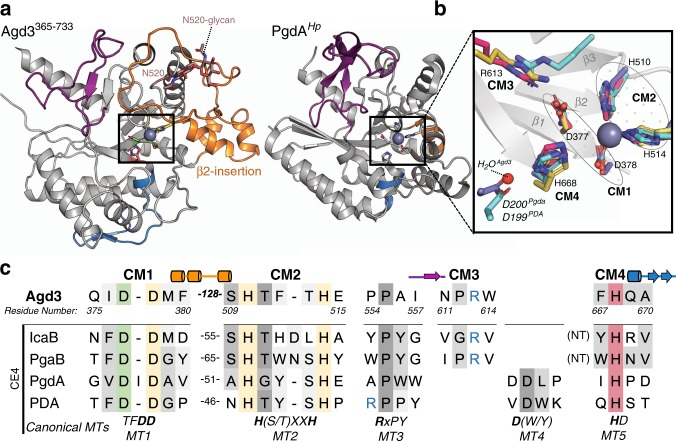
Agd3 is a unique CE enzyme with distant homology to the CE4 family. **a** Cartoon representation of the CE domain of Agd3^365–733^ (left) and PgdA (right, PDB 3QBU) with the loops between the (β/α)_7_-fold motifs colored analogously and shown in **c**. The loop after β2 (orange), β5 (purple), and the catalytic histidine (blue) are colored. The ordered sugars of the N540 glycan are in brown. **b** Structural alignment of the catalytic residues of Agd3 (pink) with four representative CE4 enzymes: *H. pylori* PgdA (3QBU, purple); *A. degensii* IcaB (4WCJ, yellow); and *B. cereus* PDA (4L1G, blue). The β-strands of the barrel are shown in gray cartoon representation. The putative catalytic motifs are labeled from CM1-CM4 and the β-stands before CM1 and CM2 have been numbered. **c** Primary sequence alignment of Agd3 catalytic motifs (CM1-4) with the CE4 MT1–MT5 as determined by structural alignment. The putative catalytic base (D377), metal coordinating triad (D378, H510, and H514) and putative catalytic acid (H668) are highlighted in green, yellow, and red, respectively. The arginine coordinating the catalytic acid is depicted in blue font. The amino acid distance between CM1 and CM2, or MT1 and MT2 in the primary sequence is listed for each protein. The canonical CE4 motifs are summarized at the bottom of the table as defined by Aragunde et al. MT5 occurs at the N-terminal to MT1–4 for the PNAG deacetylases IcaB and PgaB as denoted by the (NT) in the table.

When the entire crystal structure of Agd3 was submitted to the DALI server, no structures with similar topological arrangements were found. The most similar structure found was the peptidoglycan deacetylase PgdA (PDB 3QBU^22^) from *Helicobacter pylori*, which aligned to the putative CE-domain, with a root mean square deviation (RMSD) of 2.84 Å over 227 α-carbons. Agd3 and PgdA share only 11.9% sequence identity, and while the central (β/α)-fold is similar between these proteins, there are large differences in the regions connecting the (β/α) motifs (Fig. 3a). As PgdA does not have all the canonical CE4 motifs, we compared Agd3 with a prototypical CE4, *Bc*1960 from *Bacillus cereus* (PDB 4L1G^23^,). *Bc*1960 has 11.4% sequence identity to Agd3 and aligned with a RMSD of 2.61 Å over 175 α-carbons. A third CE4 enzyme, IcaB from *Ammonifex degensii* (PDB 4WCJ^24^) was also aligned to Agd3 due to its functional similarity. Both proteins are extracellular and IcaB is active on the biofilm exopolysaccharide, poly-β-1,6-*N*-acetylglucosamine (PNAG)^24^. IcaB has only 5.2% sequence identity to Agd3 and aligned poorly compared with the other CE4 enzymes with a RMSD of 3.80 Å over 134 α-carbons. The catalytic cations of these CE4 enzymes overlapped with a Zn ion in Agd3, suggesting that Agd3 is also metal dependent.

The structure of Agd3, analysis of its conversed residues and alignment to CE4 members enabled the identification of four catalytic motifs, CM1–4. CE4 family members have five canonical active site motifs (MT1–5)^21,25^. The MT1-5 motifs coordinate the catalytic metal and participate in the deacetylation event^21,25^. In Agd3 CM1, 2, and 4 are similar to CE4 MT1, 2, and 5, respectively. Only one of these motifs (CM2) was identifiable from sequence alignments (Fig. 3b). D377 and D378 of CM1 aligned well with CE4 MT1, which are the catalytic base and one member of the metal coordinating triad. CM2 was identified previously using sequence alignment due to its conservation with the HXXXH motif of MT2 (Fig. 3b,c). In Agd3, these histidines are H510 and H514 and each participates in metal coordination. CM1 and CM2 are separated by over 120 residues, which is more than twice the distance observed between equivalent motifs in CE4 enzymes (Fig. 3c). This difference is the result of a 64-amino acid insertion following strand β2 of the barrel, which is not present in CE4 enzymes. The 64-amino acid insertion consists of three small α-helices that constitute an extension on one side of the putative active site (β2-insertion, Fig. 3a). The β2-insertion “caps” the loops following β3 and β4 of the barrel, creating a deeper active site cleft (Supplementary Fig. 1). N520, following β3, is the location of one of the four N-glycans that could be modeled into the electron density (Fig. 3a and Supplementary Fig. 1). The β2-insertion loops around this N-glycan with T471 hydrogen bonding the O6 of the first GlcNAc, and F463 and P460 contributing to a hydrophobic pocket for the methyl of the GlcNAc (Supplementary Fig. 1).

Further differences were found between Agd3 and the other CE4 motifs. CM3 is unique to Agd3 and contains a highly conserved arginine (R613) that coordinates the putative catalytic base D377. R613 aligns structurally with the base coordinating arginine in PNAG deacetylases IcaB and PgaB (PDB 4F9D^26^). Canonical CE4 enzymes have an equivalent arginine in MT3 but this occurs much earlier in the primary sequence. Agd3 contains the conserved proline homologous to the proline of MT3 but does not have the activating arginine in this motif (Fig. 3c). The last Agd3 family catalytic motif, CM4, contains the putative catalytic acid, H668. CM4 aligns structurally with the CE4 MT5, a motif with little sequence conservation besides the catalytic histidine^21,25^. Agd3 does not contain an equivalent to the CE4 MT4, and thus lacks an aspartic acid that activates the catalytic acid in prototypical CE4 enzymes^21,25^. An ordered water molecule was found in the place of the MT4 aspartic acid (Fig. 3b), suggesting differences in mechanism between these enzymes. Despite the low overall sequence identity, the similarity of the CM1–4 catalytic motifs of Agd3 to the MT1–5 CE4 family motifs suggests that Agd3 is an active carbohydrate deacetylase that is dependent on a divalent cation cofactor. Agd3 may have a different catalytic mechanism relative to prototypic CE4 enzymes due to the lack of the activating aspartic acid, and may be more similar to PNAG deacetylases that also lack this motif^24,26^.

### Agd3 induces GAG adherence and biofilm formation

**Fig. 4:**
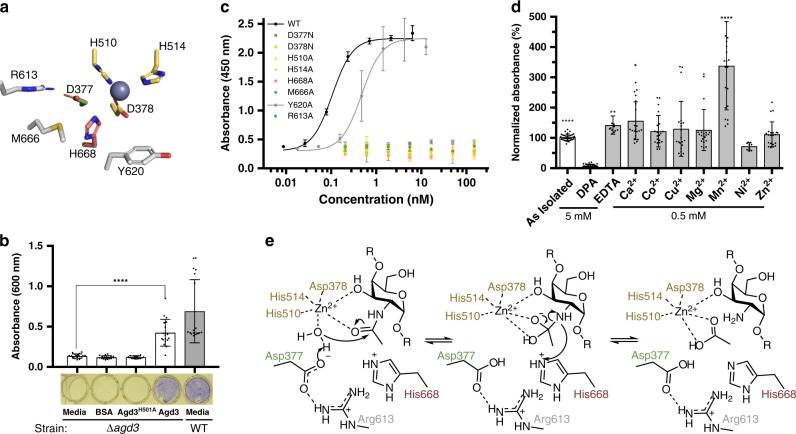
Agd3 deacetylates GAG in a metal-dependent manner. **a** Cartoon representation of the conserved active site residues including the metal coordinating triad (yellow), the putative catalytic base (green), and the putative catalytic acid (pink). **b** Absorbance of crystal violet stained 21 h old biofilms of Δ*agd3* or WT strains after treatment as labeled. Bars indicate mean ± SD and represent three independent experiments of *n* = 6. Representative stained biofilms in a 96-well plate are aligned below. **c** Agd3 deacetylation of GAG as measured by the GAG-ELLA. The experiment was run in triplicate. **d** Agd3 deacetylates GAG in a metal-dependent mechanism. Each condition had a total number of *n* = 21 for all metal conditions except Ni^2+^ with *n* = 9, *n* = 34 for as isolated, *n* = 12 for EDTA, and *n* = 28 for DPA, representing individual wells spread over nine independent experiments. **e** Metal-dependent de-*N-*acetylation reaction based on the findings of the activity assays and CE4 enzymes. The residues are color coded as in **a**. For **b** and **d** statistics were calculated one-way ANOVA with Dunnett’s multiple comparison test. *p < 0.033, ***p < 0.0002, ****p < 0.0001. Error bars represent SD. Source data is available in Source Data file.

Previously we showed that the *A. fumigatus* ∆*agd3* strain could form a biofilm when co-cultured with culture supernatants from the ∆*uge3* mutant, a GAG-deficient strain that secretes Agd3. To investigate whether there were other factors involved in this complementation we sought to determine whether recombinant Agd3 was sufficient to rescue ∆*agd3* biofilm formation. Addition of 0.5 nM recombinant Agd3 to ∆*agd3* cultures rescued biofilm formation as measured by crystal violet staining (Fig. 4b). Addition of Agd3^H510A^, an alanine mutant that would compromise metal coordination, did not complement biofilm formation suggesting that biofilm rescue was the consequence of the activity of the enzyme and that the histidine is required for activity.

To further probe the activity of Agd3 and identify catalytically important residues, an enzyme-linked lectin assay (ELLA) to detect carbohydrate deacetylase activity was developed using the native GAG substrate. The deacetylation of GAG renders the polymer adherent to plastic surfaces. Immobilized GAG was detected and quantified using the GalNAc-specific lectin Soybean-Agglutanin (SBA). In this assay, treatment of fully-acetylated GAG, from the ∆*agd3* strain, with Agd3 at concentrations as low as 80 pM resulted in detectable deacetylated adherent polymer (Fig. 4c). Mutation of the residues that coordinate the metal, H510A, H514A, or D378N, led to no detectable GAG adhesion (Fig. 4c). Similarly, mutation of the putative catalytic acid (H668A) and catalytic base (D377N) also abrogated GAG adhesion even when the enzyme concentration was increased 1875-fold, to 150 nM (Fig. 4c). The active site proximal arginine, R613 found in CM3, is also integral for activity in this assay. M666 in CM4 is highly conserved in Agd3 homologs and when mutated to alanine also abrogates adhesion suggesting the importance of this residue in the formation of the active site and/or substrate alignment.

Aromatic residues are often found in and near the active sites of carbohydrate active enzymes^21,24,27–29^. Y620 is near the active site and could potentially be involved in substrate binding. Mutation of Y620 to alanine led to a minor decrease in activity with a 50% effective concentration (EC_50_) of 0.48 nM (95% confidence interval [CI] of 3.3–7.0 nM) compared with 0.11 nM (95% CI of 0.10–0.13 nM) for the wildtype protein under these conditions (Fig. 4c).

To determine whether Agd3 was metal dependent and the identity of the metal, Agd3 was incubated with chelators or the chloride salts of various divalent cations before addition of the GAG substrate. Addition of dipicolinic acid (DPA) abolished Agd3 activity whereas EDTA had no significant effect (Fig. 4d). Under the experimental conditions tested, exogenous addition of Mn^2+^ resulted in almost threefold increase in activity as compared with non-treated Agd3 (Fig. 4d). Combined, these results suggest that Agd3, similar to CE4 family members^21^, uses a divalent cation to stabilize a tetrahedral intermediate (Fig. 4d).

### Agd3 de-*N*-acetylates α-1,4-GalNAc oligos with length dependence

**Fig. 5:**
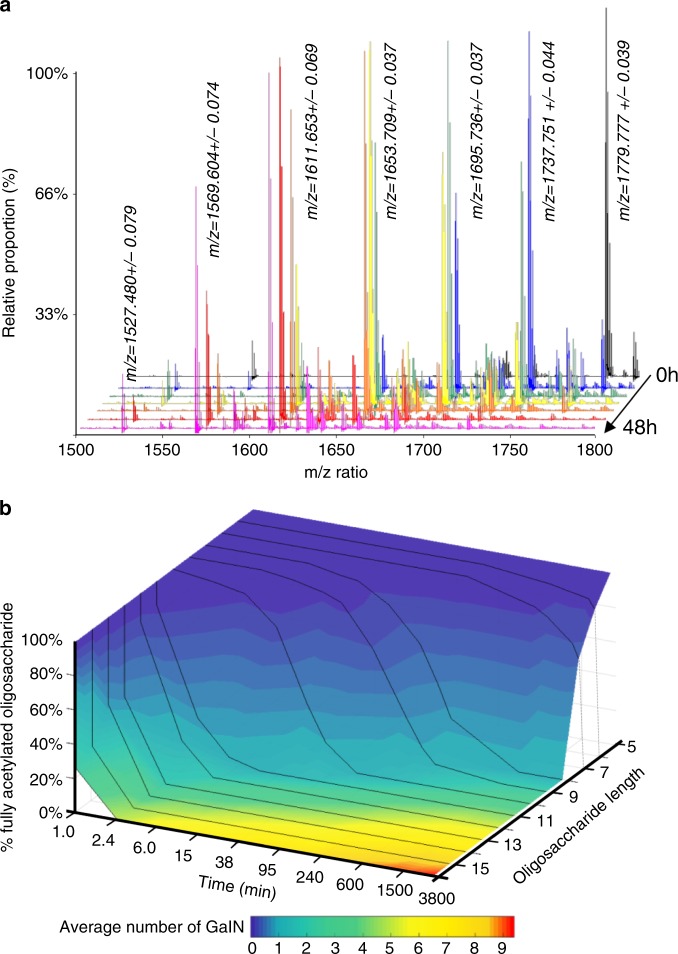
Effects of substrate length on the de-*N*-acetylation activity of Agd3. **a** Time course of α-1,4-(GalNAc)_8_ de-*N*-acetylation by 1 µM Agd3 over 48 h. Enzymatic activity was monitored by MALDI-TOF MS of the *m*/*z* window 1500– 1800. The MS traces correspond to the following time points: 0, 1, 2, 3, 6, 24, and 48 h. **b** Time course of de-*N*-acetylation of GalNAc oligosaccharides ranging from pentamer to 16-mers. Signals from each oligosaccharide were integrated and graphed as the percentage of fully acetylated oligosaccharide over time. The degree of de-*N-*acetylation of each oligosaccharide is indicated by the color of the 3D surface.

To confirm the de-*N*-acetylation activity of Agd3 at the molecular level, the activity on synthetic α-1,4-(GalNAc)_8_ was analyzed by matrix-assisted laser desorption/ionization time of flight (MALDI-TOF) mass spectrometry (MS). Consistent with the results of the GAG-ELLA and biofilm assays, Agd3 treatment of the octasaccharide resulted in the progressive loss of an average *m*/*z* ratio of 42.014 ± 0.005, corresponding to the monoisotopic mass of an acetate group (Fig. 5a). Agd3 treatment at concentrations as high as 10 µM had no effect on chitin and chitosan oligosaccharides (ß-1,4-*N*-acetylglucosamine and partially deacetylated ß-1,4-*N*-acetylglucosamine, respectively), suggesting that Agd3 deacetylation activity is specific for GalNAc (Supplementary Fig. 2).

To determine the effect of substrate length on enzyme activity, Agd3 was incubated with a pool of GalNAc oligosaccharides of varying lengths (5–16 units) that were isolated from *A. fumigatus* biofilms (Supplementary Fig. 3). Examination of the rate and extent of de-*N*-acetylation of the pool of oligosaccharides following Agd3 treatment revealed a direct correlation between the length of the oligosaccharides and the rate of de-*N*-acetylation (Fig. 5b). GalNAc oligosaccharides shorter than seven units were poorly deacetylated, even after 48 h of treatment. In contrast, GalNAc oligosaccharides longer than 13-mers were rapidly deacetylated by Agd3 multiple times within 5 min (Fig. 5b). The presence of deacetylated GalN moieties within oligosaccharides did not seem to impair subsequent de-*N*-acetylation events, suggesting that Agd3 can bind oligosaccharides containing both GalNAc and GalN. The direct evidence by MS that Agd3 is an active carbohydrate deacetylase, with activity on α-1,4-GalNAc substrates, and the low sequence identity to any current CE family, led to the creation of the CE18 family.

### Agd3 contains a unique carbohydrate binding module (CBM)

**Fig. 6:**
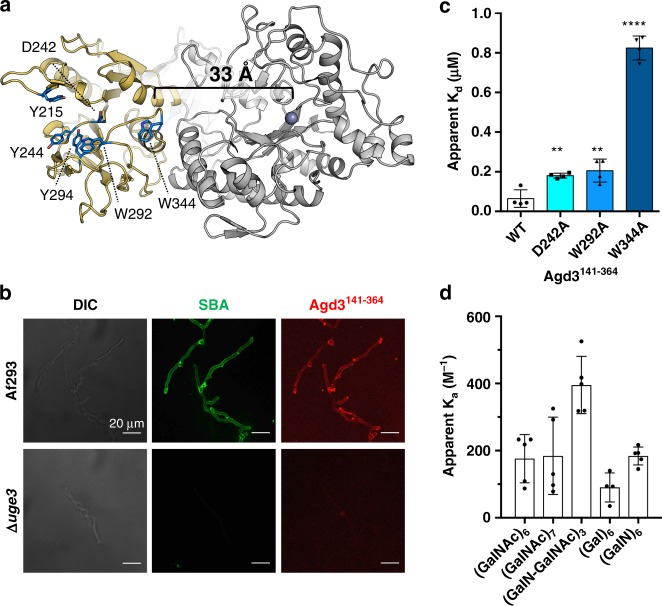
Agd3 contains a unique carbohydrate binding module. **a** The structure of Agd3 showing the CBM in yellow with the first conserved tryptophan (W344) ~33 Å from the active site. **b** WT (Af293) and ∆*uge3* hyphae visualized by differential interference contrast (DIC, left) and fluorescent microscopy co-stained with SBA (center, green) and Alexa-568 labeled Agd3^141–364^ (right, red). Representative images from two independent experiments each of three or more images for each condition. Recombinant Agd3^141–364^ localizes to the hyphal surface in a GAG dependant manner similar to SBA. **c** Apparent *K*_d_ (µM) for Agd3^141–364^, and point mutants thereof, to soluble GAG as determined using GAG-ELISA. Bars represent mean *K*_d_ values ± one SD of four experiments run in triplicate. Statistics were calculated using one-way ANOVA with Dunnett’s multiple comparison test. *p < 0.013, **p < 0.0034, ****p < 0.0001. **d** Apparent *K*_a_ (M^−1^) for Agd3^141–364^ for α-1,4-linked carbohydrate ligands determined by a direct ESI-MS assay. Experiments were performed at 25 °C and pH 7.2 with *n* = 4 for (Gal)_6_ and *n* = 5 for all others. Each data point represents the calculated *K*_a_ value from a single ESI-MS assay. Bars are the mean ± one SD. Source data is available in Source Data file.

The results of the oligosaccharide deacetylation studies demonstrated that Agd3 prefers longer substrates. These findings suggest a long binding cleft that provides increased affinity for the longer polymers over shorter ones. Investigation of the structure found that the cleft of the α/β/α-domain (Agd3^141–364^) aligns with the cleft of the CE-domain (Fig. 6a). The α/β/α-domain cleft is lined with surface exposed conserved aromatic residues, which are commonly involved in carbohydrate binding^27,29–32^ (Fig. 6a).

The N-terminal domain of Agd3 (141–364) forms a seven-stranded parallel β-sheet that is sandwiched between three α-helices, one helix on one side (α2) and two on the other (α1 and α4) (Supplementary Fig. 4a). According to the DALI server, Agd3^141–364^ is most structurally similar to two uncharacterized class 1 glutamine synthase (GATase1)-like proteins: GATase1-like protein from *Planctomyces limnophilus* (PDB 3RHT); and the ThuA-like protein from *Bacteroides uniformis* (PDB 4JQS). Secondary structure alignment revealed the shared topology with RMSD of 2.63 and 2.73 Å over 176 α-carbon atoms for GATase1- and ThuA-like proteins, respectively (Supplementary Fig. 4b). The ThuA-like protein has higher sequence identity to Agd3^141–364^ (15.9%) than to GATase1 (10.2%). Agd3 does not share conserved surface residues with either of these proteins, nor the catalytic GATases Cys–His–Glu triad, all of which are located on the C-terminal end of the central β-sheet^33^. Instead Agd3 and homologs have conserved surface exposed aromatics in this region (Fig. 6a).

To examine whether the N-terminal Agd3^141–364^ domain serves as a CBM in catalysis, GAG binding assays were performed. We found that recombinant fluorescently-labeled Agd3^141–364^ co-localized with SBA on hyphae surfaces (Fig. 6b). No Agd3^141–364^ was detected on the surface of the GAG-deficient ∆*uge3* strain (Fig. 6b), suggesting that Agd3^141–364^ binds to cell-associated GAG. Agd3^141–364^ also bound to soluble GAG in a dose-dependent manner. Mutation of conserved residues within the cleft, D242, W292, or W344, to alanine increased the apparent dissociation constant (*K*_d_) for soluble GAG binding, suggesting that this region plays a role in this process (Fig. 6c). W344A point mutation had the largest effect, with a *K*_d_ 12.7-fold higher than the wild-type protein.

Ligand specificity of the CBM was explored using synthesized GAG oligosaccharides. Association constants (*K*_a_) were determined by direct electrospray ionization MS (ESI-MS) for each oligosaccharide. Agd3^141–364^ bound α-1,4-(GalNAc)_6_ and α-1,4-(GalN)_6_ with *K*_a_ of 180 ± 60 M^−1^ and 180 ± 30 M^−1^, respectively, (Fig. 6d). No statistically significant difference was found between the binding of (GalNAc)_6_ and (GalNAc)_7_, suggesting that the binding site spans six or fewer subsites, which is consistent with the length of the identified cleft (Fig. 6d). Agd3^141–364^ binding of (Gal)_6_ was negligible, suggesting that the CBM of Agd3 is specific for regions of the GAG polymer that are GalNAc/GalN rich. Interestingly, there was slightly, but significantly, higher affinity for a mixed GalN-GalNAc oligosaccharide (*K*_a_ 400 ± 90 M^−1^, Fig. 6d). This finding suggests that partial deacetylation of the polymer could lead to higher affinity, and hence accelerated deacetylation after the initial deacetylation events have occurred.

### The CBM augments Agd3 deacetylation

**Fig. 7:**
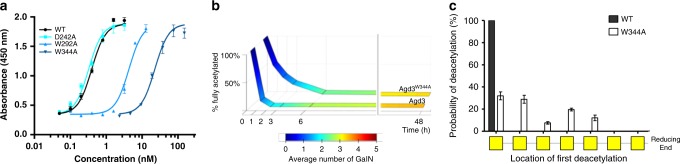
Agd3 CBM increases deacetylation rate and specificity. **a** Deacetylation of GAG by Agd3 or point mutants as determined by GAG-ELLA. Experiment performed in triplicate and EC_50_ calculated from three independent experiments. One representative experiment is data shown as mean values ± one SD. **b** Time course of α-1,4-(GalNAc)_7_ deacetylation by Agd3 or Agd3^W344A^. Products were analyzed by MALDI-TOF MS and graphed as the relative amount of unmodified substrate. The average number of deacetylation events is indicated by the color legend. **c** Analysis of the MALDI-TOF MS-MS fragmentation of mono-deacetylated heptasaccharide product produced Agd3 or Agd3^W344A^. The *X*-axis indicates the location of the deacetylation event within the heptasaccharide. Mean values are of 11 experiments and error bars represent SD. Source data is available in Source Data file.

To determine the role of the CBM in modulating the activity of Agd3, the deacetylase activity of the W292A and W344A mutants were compared with that of recombinant wild-type Agd3. Agd3 constructs lacking the CBM, with and without the C-terminal domain, were unstable so activity of the CE domain alone could not be determined. Mutation of W292 or W344 to alanine resulted in a significant decrease in GAG deacetylation as measured by the GAG-ELLA. When compared with the wild-type enzyme, Agd3^W292A^ and Agd3^W344A^ exhibited a 13-fold (4.3 ± 0.29 nM) and 58-fold (19.8 ± 1.89 nM) higher EC_50,_ respectively, suggesting that these sites may influence substrate positioning and/or affinity in the context of the multidomain enzyme (Fig. 7a). As W344 is the predicted CBM binding subsite closest to the active site, and had the most dramatic effect on deacetylase activity when mutated, this mutant was selected for further studies to determine the role of the CBM in substrate binding and de-*N-*acetylation.

To study the difference between the de-*N-*acetylation activity of wild-type Agd3 and Agd3^W344A^ in detail, synthetic α-1,4-GalNAc heptasaccharides and octasaccharides were treated with both enzymes, and the reaction products quantified over time by MALDI-TOF MS. Initial deacetylation of α-1,4-(GalNAc)_7_ by Agd3^W344A^ was significantly slower than by wild-type Agd3 (Fig. 7b). Conversion of α-1,4-(GalNAc)_7_ by Agd3^W344A^ into >97.5% (GalNAc)_6_GalN required over 6 h, as compared with two hours for the wild-type Agd3 (Fig. 7b). The rate of subsequent deacetylation events was not different between the two enzymes (Fig. 7b). Similar results were obtained when the synthetic GalNAc octasaccharide was used as a substrate (Supplementary Fig. 5).

The W344A mutation also resulted in a change in the position of the primary de-*N-*acetylation event within the oligosaccharide substrate (Fig. 7c and Supplementary Fig. 6). The primary deacetylation event in wild-type Agd3 was observed exclusively at the GalNAc located at the non-reducing end of the oligosaccharide. In contrast, Agd3^W344A^ treatment resulted in deacetylation of one of the five GalNAc located at the nonreducing end of the oligosaccharide (Fig. 7c). Subsequent deacetylation events by both enzymes occurred at one of the four remaining GalNAc located at the nonreducing end of the oligosaccharide. Neither enzyme was able to modify the two GalNAc residues proximal to the reducing end (Supplementary Fig. 6). The decrease in deacetylation rate and altered specificity of the first deacetylation event in the W344A mutant suggests that the CBM aids in substrate alignment within the active site and thus more efficient, and specific, binding of oligosaccharides.

### Agd3 homologs are present in bacterial and fungal species

**Fig. 8:**
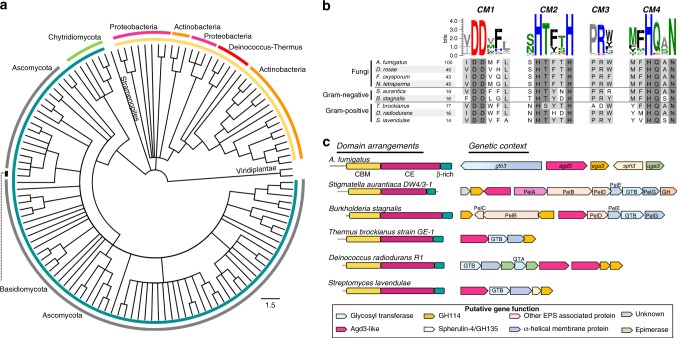
Agd3 homologs are present in bacterial and fungal kingdoms. **a** The maximum-likelihood phylogeny tree constructed using MEGA7 containing 126 representative sequences of the BLASTP results. Visualization was done in FigTree with a bootstrap cutoff of 0.7 of 250 iterations. Bacterial sequences are highlighted in yellow and fungal in teal. **b** Sequence logo (created with WebLogo 3.6.0) of the four catalytic motifs (CM1–4) calculated from the 126 sequences used in **a**. The logo is accompanied by the amino acid sequence alignment of representative *agd3* homologs. Proteins from *Diplocarpon rosae, Sclerotinia sclerotiorum*, and *Neurospora tetrasperma* were used as Agd3 orthologues. Bacterial proteins were aligned and are the same as those shown in **c**. Sequence identity is shown for each as calculated by the final alignment done by MUSCLE in Geneious. **c** Linear domain arrangement and genetic location of each of the five-representative bacterial Agd3 homologs. Domains are shown to scale. Bacterial operons are to scale relative to each other but not to the GAG-cluster, due to intronic DNA. The list of organisms used in the tree is available in the Supplementary Data 2.

Agd3 orthologues were previously found across the *Ascomycota* phylum, located within gene clusters resembling the GAG biosynthetic gene cluster of *A. fumigatus*. Broadening this search to the nonredundant protein database using JackHMMer and BLASTP over 800 sequences with >20% identity were found. Agd3 homologs were found in 311 different species, including in 266 eukaryotic and 45 bacterial species. The majority of high confidence hits were in the *Ascomycota* phylum as previously determined, as well as in five fungal species within the *Chytridiomycota* phylum including anaerobic herbivore symbiotes and one aquatic fungi, *Gonapodya prolifera*. Agd3 homologs were found in 30 Terrabacteria, including *Actinobacter* and *Deinococcus-Thermus* spp. (Supplementary Data 1). Gram-negative hits were found in Proteobacteria such as *Bulkholderia*, *Moritella*, and *Stigmatella*. After removing sequences with over 80% identity and those that were protein fragments, 126 sequences were selected and used to generate a maximum-likelihood tree (Fig. 8a). This tree revealed separate clustering of the bacterial and eukaryotic sequences suggesting horizontal gene transfer may have occurred between ancestors of these clades.

Alignment of the Agd3 homologs to Agd3 showed high conservation of all four CMs (Fig. 8a). CM3 is conserved in all the fungal enzymes and in most of the bacterial homologs. Bacterial genes had homology spanning all three of the globular domains of Agd3 (Fig. 8b). The C-terminal domain had the lowest sequence identity, but the secondary structure predictions were consistently β-rich in character. The CBM fold was found in all hits with significant conservation of the identified aromatic residues, including over 79% similarity at W334 and 88% similarity at W292. The bacterial Agd3 homologs share low sequence identity to Agd3 (13–19% MUSCLE, 20–28% BLASTP) but the presence of the catalytic motifs and the three-domain configuration suggests that they are CE enzymes related to Agd3.

In *Ascomycota*, the *agd3* gene is encoded within the putative GAG biosynthetic gene clusters of these organisms, implying a role for Agd3 in exopolysaccharide production in these fungi^5^. There is more variation in the genetic environment of the bacterial *agd3* genes. Of the 35 bacterial species represented in the tree, 30 contain *agd3* genes located in or near operons that encode predicted carbohydrate active enzymes (Fig. 8c). There is variation in operon synteny and content between bacterial species, but the genes have similar predicted functions to those found in the GAG gene cluster (Fig. 8c). Of the operons identified, all contain genes encoding a cytoplasmic glycosyltransferase and a multi-spanning α-helical integral membrane protein. The co-occurrence of a GH114 encoding gene, homologous to *ega3*, is highly consistent throughout both Gram-negative and -positive organisms and, in some cases, multiple such genes were found in or adjacent to the operon (Fig. 8c). In *A. fumigatus, agd3* and *ega3* are found adjacent in the genome (Fig. 8c) and are coregulated^5^. Recently, Ega3 was found to be specific for deacetylated regions of GAG supporting that *agd3* and *ega3* homologs are functionally linked^9^. Spherulin-4 annotated genes were also found in some of the *Actinobacteria* operons. The Gram-negative operons were more complex and contained genes with homology to the outer membrane components of the Pel polysaccharide biosynthetic machinery (Fig. 8c). These findings support that Agd3 is the first member of a larger family of carbohydrate deacetylases that span both bacterial and fungal species.

## Discussion

In this study, we show that Agd3 is a carbohydrate deacetylase that defines a new family of carbohydrate esterases, CE18. Characterization of N-terminal domain also established a CBM family, CBM87. At present, there are 16 carbohydrate esterase families. Family CE10 has been withdrawn as the members of this family do not act on carbohydrate substrates. Only one new family has been created since 2008^34^. Agd3 has distant homology to members of the CE4 family, which includes the PNAG deacetylases, IcaB and PgaB, involved in biofilm formation in multiple Gram-positive and Gram-negative bacterial species^13,14,26,35^.

The crystal structure of Agd3 revealed a unique three-domain architecture not found in any structurally characterized protein to-date. The peptidoglycan deacetylase, PgdA (3QBU) has the closest structural similarity to Agd3, but this protein only aligns with the central CE domain of Agd3 (Fig. 3a). This structural alignment revealed commonality in active site motifs between Agd3 and the CE4 family despite the low sequence identity (11.9%) to PgdA. Agd3 has four highly conserved CMs that are essential for GAG deacetylation in vitro. Agd3 does not have a motif equivalent to MT4 in CE4 enzymes, which contains the catalytic acid activating aspartic acid. Instead, Agd3 has a water molecule in its place (Fig. 4b). The PNAG deacetylases IcaB and PgaB also lack this activating aspartate residue and have similarly structured water molecules^26^. In PgaB, the absence of the MT4 aspartic acid is proposed to be responsible for the enzyme’s lower catalytic efficiency, which correlates with the low levels of PNAG deacetylation observed in vivo^26^. Secreted *E. coli* PNAG is 22%^15^ deacetylated, or less^36^, whereas other CE4 substrates, peptidoglycan and chitin, are typically >80% deacetylated^37,38^. The degree of deacetylation of *A. fumigatus* GAG is not known, however, analysis of the GAG from *Neurospora crassa* found that the polymer has between 30 and 70% deacetylated galactosamine depending on the growth conditions. In *N. crassa*, deacetylation was correlated with the level of expression of a deacetylase^39–41^. Recently, it was shown that *Aspergillus oryzae* produces GAG with a degree of deacetylation near 50%^42^. Picomolar quantities of Agd3 are sufficient to cause soluble GAG adhesion in the GAG-ELLA assays suggesting that Agd3 is efficient, or that low levels of deacetylation are sufficient for adherence.

Agd3 has a conserved arginine, R613 in CM3, that coordinates the catalytic base, and is important for activity. This arginine is found much later in the primary sequence than the base activating arginine in MT3 of canonical CE4s^43^. However, R613 aligns structurally to the activating arginine found in IcaB, suggesting that it could play a similar role in GAG deacetylation. IcaB and other PNAG active CE4s, including PgaB from *E. coli*, contain an arginine separate from MT3 (Fig. 3c) which is more similar to Agd3 than canonical CE4s. Agd3 has catalytic motifs in the same order within the primary sequence as equivalent motifs of the CE4 family. The structural similarity results and equivalent order of CMs in the primary sequence suggests that Agd3 is distantly related to the CE4 family and may have diverged from a common ancestor.

Agd3 activity is metal-dependent as complete inhibition could be achieved with the addition of DPA (Fig. 4d). In contrast, addition of 12,500-fold molar excess EDTA did not inhibit Agd3 activity. The discrepancy between inhibitor effects can be explained by their differing modes of action. DPA has been reported to create a ternary complex with the bound metal, thus metal diffusion would not be a limiting step in inhibition^44,45^. EDTA is a larger molecule and diffusion of the metal from the metalloenzyme is required for sequestration in some cases^44^. The inability of EDTA, compared with DPA, to inactivate enzymes has also been reported for PgaB and the chitin deacetylase CDA from *Collectotrichum lindemuthianum*. These results suggest a low diffusion rate of the metal ion from the Agd3 active site. The crystal structure contained a Zn^2+^ ion but addition of Mn^2+^ to the assay conditions significantly increased Agd3 activity (Fig. 4d). It is possible that Agd3 uses a different metal in vivo. Many CE4 enzymes are Zn^2+^ dependent but some have been found to be promiscuous. The biofilm state is often nutrient limited so metal promiscuity could be beneficial.

The similarity between the active site of Agd3 and that of the CE4 members supports a shared mechanism between the CE families (Fig. 4e). For Agd3, the proposed mechanism starts with the metal ion coordinating a water molecule that is deprotonated by D377 and attacks the acetate group creating a tetrahedral oxyanion intermediate. H668 can then donate a hydrogen to break the bond between the acetate leaving group and amine (Fig. 4e).

In addition to the central CE, Agd3 has two auxiliary domains. The N-terminal α/β/α-domain was found to have some structural similarly to the glutamine-transferase I family. Our findings support that Agd3^141–364^ is a CBM capable of binding GAG. This domain does not belong to any of the current 86 families and is the first characterized CBM with a reductase-like fold^30^. CBMs are commonly found linked to glycoside hydrolases and increase the specificity and affinity of the enzyme for the substrate^30,46^. In some cases, CBMs can modulate the structure of the substrate allowing for binding by the active domain^30^. Some CE4 enzymes have been reported to have CBMs^47^. The activity of the *Podospora anserine* chitin de-*N*-acetylase on insoluble chitin was reduced by removal of one or both CBMs, although activity against soluble chitin was unaffected^48^. GAG is heterogeneous in composition and can be highly insoluble^4–6^. The Agd3 CBM may assist in binding of insoluble substrate and alignment of GAG within the active site, thus aiding in the solubilization of the acetylated polymer.

Phylogenetic analysis found that the three-domain architecture of Agd3 is present in both fungal and bacterial kingdoms. In contrast to the members of the CE4 family which do not have consistent domain arrangement, the CBM domain is present in the majority of the Agd3 homologs found^21^. Fungal Agd3 orthologues have been identified previously within GAG gene clusters^5^. The production of GAG-like polymers has been confirmed in *Aspergillus niger*, *Aspergillus parasiticus*, *A. oryzae*, *N. crassa*, *Penicillium frequentans*, *Paecilomyces* sp., and *Trichosporon asahii*^5,40,42,49–53^. Expression of poly-*N*-acetylgalactosamine deacetylases in both *A. parasiticus* and *N. crassa* has been confirmed and enzyme levels are linked to levels of polysaccharide produced suggesting co-regulation of polymer and deacetylase production^40,54^. In this study, we found Agd3 homologs in bacteria encoded within, or near, operons containing genes of analogous function to those of the GAG cluster. Some Gram-negative Agd3 homologs were located near genes with homology to Pel polysaccharide genes. Pel, like GAG, is a partially de-*N*-acetylated GalNAc-rich polymer involved in biofilm formation in some *P. aeruginosa* strains^55–58^. Pel operons have been found in *Proteobacteria*, including *Burkholderia* and *Moritella* species^59^ and more recently in a wide range of Gram-positive bacteria^60,61^, suggesting that many bacteria produce a Pel/GAG-like polymer.

The synteny and composition of *agd3*-containing operons in Gram-positive organisms was variable. Many of the organisms identified are soil-dwelling and plant pathogens, thus inhabiting similar environments as the *Ascomycota* species. Low levels of galactosamine-containing polysaccharides have been found in the cell-walls of *Streptomyces* species^62,63^. Expression and secretion of an active endo-α-1,4-galactosaminidase has also been reported in *Streptomyces griseus*, suggesting that this species produces or encounters a GalNAc-rich polymer, similar to GAG or Pel^64^. Previously, it had been suggested that the GAG gene cluster had been horizontally transferred from bacteria to a common ancestor of *Pezizomycotina*, due to the clustered nature of the genes and the similarity to bacterial synthase-dependent exopolysaccharide biosynthetic systems^5^. Our findings of operons containing most, or all, of the genes homologous to the GAG cluster adds further support to this hypothesis.

Agd3 is required for GAG maturation and full virulence in the mouse model of *A. fumigatus* infection. Structural and functional analysis of the domains within Agd3 have identified novel CE family, CE18, and CBM family, CBM87, domains. Our findings suggest that there are many Agd3 homologs within both fungal and bacterial species. Further experiments to determine the function and importance of Agd3 homologs will aid in our understanding of exopolysaccharide modification systems across kingdoms.

## Methods

### Aspergillus strains and growth conditions

The wild-type *A. fumigatus* strain Af293 and Af293 with an *agd3* knockout (Af293 *∆agd3*) produced by Lee et al.^5^ were used in this study. Unless otherwise noted, strains were grown and harvested on YPD agar (Fisher Scientific) at 37 °C as previously described^65^. For growth in liquid medium, Brian medium^4^, AspMM^66^, and RPMI media 1640 (Wissent) were used as indicated. All strains and primers used in this study are summarized in Supplementary Table 2.

### Bioinformatics analysis

The Agd3 sequence from *A. fumigatus* was analyzed using Phyre^2^^18^ and NetOGlyc 4.0 server^19^. Sequence alignment was done using Clustal Omega^67^ and MUSCLE^68^.

### Agd3 expression and purification

*A. fumigatus* Af293 *agd3* (Afu3g07870), was codon optimized for *P. pastoris* (Bio Basics Inc.) and received in pUC57 plasmids (pUC57-Agd3Pp). The region of pUC57-Agd3Pp encoding residues 141–806 (referred to as Agd3 herein) was cloned into a pET28a vector. This gene, with the addition of the N-terminal histidine tag and thrombin cleavage site encoded by the pET28a vector, was subsequently cloned into the pPinkα-HC plasmid for expression in the PichiaPink^TM^ secretion system (Invitrogen). Expression was induced with 1% (v/v) methanol for 16–24 h. Culture supernatants were filtered and the protein purified by precipitation with 65% (v/v) ammonium sulfate, dialysis, and size exclusion chromatography. Point mutants were expressed and purified as described above.

Agd3^141–364^, and point mutants thereof, were expressed and purified from *E. coli* Origami2 cells using the pET28a vector. Purification was performed using nickel affinity and size exclusion chromatography.

### Crystallization and structural determination

Purified Agd3 was screened for crystallization conditions using sitting-drop vapor diffusion at 6 and 10 mg/mL. Crystals formed in 1.7 M ammonium sulfate, 1.7% (v/v) PEG 400, 85 mM HEPES pH 7.5, and 15% (v/v) glycerol (Rigaku TOP96 condition #8). Optimization through additives and streak seeding produced crystals in TOP96 #8 with 0.25% (v/v) PEG 3350. Two crystals were harvested and soaked in 1.36 M ammonium sulfate, 260 mM zinc sulfate, 85 mM HEPES pH 7.5, 2.36% (v/v) PEG 400, 14% (v/v) glycerol before vitrification in liquid nitrogen. Data were collected at the NSLS II using the Frontiers Macromolecular Crystallography beamline at −173 °C and a wavelength of 1.283 Å. Data sets were merged using XPrep and the structure solved using the SHELX suite^69,70^. SHELXE found six unique zinc ions with significant anomalous signal and was able to build poly-alanine traces for 315 amino acids. Residues 141-806 were built into initial maps using Coot through SBGrid^71–74^. There was no interpretable density for the hexahistidine tag. Maps were converted to .mtz format using F2MTZ through CCP4^75–77^ and the refinement performed using PHENIX.REFINE with iterative real-space refinement in Coot. Once a model was determined with residual values in the low 20s, this model was used for molecular replacement against the highest resolution single data set. The anisotropy of this single dataset was corrected using the Diffraction Anisotropy Server^78^. Model refinement was done in real space in Coot and through PHENIX.REFINE. TLS groups were added with the TSLMD server in PHENIX^71,72^.

### Rescue of biofilm formation by addition of Agd3

Af293 Δ*agd3* and Af293 strains were cultured in Brian media with or without supplementation of BSA protein, Agd3^H510A^, or Agd3 wildtype for 21 h in 96-well round-bottom tissue-culture treated plates. Protein supplements were added at a final concentration of 0.5 nM. Biofilms were washed with deionized water, stained with 0.1% (w/v) crystal violet for 10 min and destained with 100% ethanol for 10 min. Absorbance of destain solution was measured at 600 nm. For biofilm imaging, Δ*agd3* and Af293 were cultured for 27 h in a 24-well flat bottom non-tissue-culture treated plate which was washed with PBS and stained with crystal violet.

### GAG deacetylation assay

A GAG-ELLA was used to assess the activity of Agd3^141–806^ WT or its point mutants. Briefly, 50 µL of protein, in PBS pH 7.4, was mixed with 50 µL of culture supernatants harvested from the Af293 ∆*agd3* strain^5^ in a 96-well clear flat-bottom Immulon 4HBX plate (Thermofisher). Reactions were incubated at room temperature for 1 h on a mutator. Plates were washed with PBS-T three times before the addition of 100 µL of lectin solution (30 nM biotinylated soybean agglutinin [SBA-biotin, Vector Labs], 1/700 avidin horseradish peroxidase [Avidin-HRP, Invitrogen]). After incubation at room temperature for 1 h, plates were washed with PBS-T three times followed by addition of 100 µL of TMB Ultrasensitive Solution (Millipore). Plates were read at 370 nm or the reaction was stopped with 100 µL of 2 M H_2_SO_4_ and read at 450 nm. For inhibitor and metal screening, chloride salts, EDTA, or DPA were incubated with the protein for 10 min before the addition of the culture supernatant.

### MALDI-TOF MS enzymatic fingerprint

α-1,4-GalNAc oligosaccharides were obtained by a Sph3 partial hydrolysis. *A. fumigatus* biofilms were incubated with 5 nM Sph3 for 1 h at room temperature, solubilized oligosaccharides were then further purified on a Sep-pak C18 cartridge. In brief, cartridges were conditioned using absolute ethanol followed by water. Samples were then loaded onto the cartridge before washing and eluting using a 1% (v/v) step gradient of ACN from 1 to 4% (v/v). 3% (v/v) ACN fraction was used for the specificity study.

Purified oligosaccharide fractions were mixed with Agd3 WT or its mutants as indicated, and the samples analyzed by MS at multiple time points. Products of enzymatic reaction were diluted in 0.2% (v/v) TFA before being spotted on the MALDI-TOF plate in a ratio 1:1 (v:v) with 5 mg/mL DHB matrix reconstituted in ACN: 0.2% (v/v) TFA (70:30, v–v). Spectra were recorded on a Bruker UltrafleXtreme in positive reflector mode and an accumulation of 5000 laser shots. MALDI-TOF MS/MS experiments were performed using the same mass spectrometer. Data was collected and analyzed using Bruker Flex Control v.3.4 and Bruker Flex Analysis v.3.4.

### Investigation of Agd3 specificity

Oligosaccharides of chitin were produced by acidic partial hydrolysis of chitin from shrimp shell (Sigma). Briefly, chitin was incubated in 0.1 M HCl for 3 h at 100 °C. Solubilized oligosaccharides of chitin were then re-*N*-acetylated by incubation in methanol: pyridine: anhydride acetic (10:2:3) for 1 h at room temperature. Oligosaccharides were then purified on a Hypercarb SPE cartridge (Thermofisher) conditioned as per manufacturer instructions. After loading the sample, the cartridge was washed with water, 5% (v/v) ACN, and oligosaccharides eluted with 50% (v/v) ACN.

Purified chitin oligosaccharides were then incubated with 10 µM Agd3 in 1X PBS for 24 h. Results of the incubation were then spotted on the MALDI-TOF plate in a ratio 1:1 (v:v) with 5 mg/ml DHB matrix reconstituted in ACN: 0.2% (v/v) TFA (70:30, v–v). Spectra were recorded on a Bruker UltrafleXtreme in positive reflector mode and an accumulation of 5000 laser shots. Data were collected and analyzed with Bruker Flex Control v.3.4 and Bruker Flex Analysis v.3.4.

### Fluorescence microscopy of *A. fumigatus* hyphae

Hyphae were grown as above on tissue culture treated cover slips, washed twice with PBS and stained with 120 μg/ml of the GalNAc specific lectin Soybean Agglutinin lectin (SBA) conjugated to fluorescein and 10 μM of Alexa Fluor^®^−568 labelled Agd3^141-364^ for 2 h, at 4 °C. Samples were washed twice with PBS and fixed with 4% (w/v) paraformaldehyde for 20 min at 4 °C. Samples were washed twice with PBS and mounted with SlowFade^®^ mounting media and sealed. Samples were imaged on a Zeiss confocal microscope with 488 and 561 nm lasers to excite fluorescein and Alexa Fluor®-568, respectively. The channels were acquired separately, with fluorescein being detected from 490–577 nm and Alexa Fluor^®^-568 from 577 to 691 nm.

### GAG binding assay

Undiluted culture supernatants were incubated in Immulon 4HBX 96-well microtiter plates (Thermofisher) overnight at 4 °C. Wells were washed 3× with wash buffer [PBS, 0.05% (v/v) Tween-20] and blocked for 1 h in blocking buffer [2% (w/v) BSA in wash buffer]. Wells were washed 1× with wash buffer and incubated with the indicated concentrations of Agd3 diluted in blocking buffer for 2 h at room temperature. Wells were washed 3× with wash buffer and incubated with mouse anti-His-Tag (Abgent) diluted 1/1000 in blocking buffer for 45 min. Wells were then washed 3× with wash buffer and incubated with donkey anti-mouse secondary antibody (Cedarlane) conjugated to horseradish peroxidase diluted 1/1000 in blocking buffer for 45 min. Wells were then washed 4× with wash buffer and incubated with TMB substrate (ThermoFisher) for 6 min at room temperature. The reaction was stopped with the addition of 1 M H_2_SO_4_ and absorbance read at 450 nm.

### Synthesis of α-1,4-linked oligosaccharides

Gal and galactosamine oligosaccharides were synthesized as described previously^8^. This method was also used to generate *N*-acetylgalactosamine and mixed *N*-acetylgalactosamine/galactosamine oligosaccharides. Briefly, oligosaccharides were chemically synthetized using building blocks protected with di-*tert*-butylsilylene. These cis-galactosylating agents are extremely selective and only produced α-isomer products. After glycosylation reactions, the di-*tert*-butylsilylene group was removed with HF pyridine, and the free hydroxyl was protected with benzoyl group. Through a linear stepwise synthesis strategy, the following oligosaccharides were made: α-1,4-(Gal)_6_, α-1,4-(GalNAc)_6-8_, α-1,4-(GalN)_6_, and α-1,4-(GalN-α-1,4-GalNAc)_3_. Finally, deprotection by saponification, debenzylation, azide reduction and acetylation of amine groups was preformed to obtain the oligosaccharides. These compounds have been characterized with ^1^H-nuclear magnetic resonance (NMR), ^13^C-NMR, HH-correlation spectroscopy, heteronuclear single quantum coherence spectroscopy, and high resolution MS.

### Electrospray ionization mass spectrometry (ESI-MS)

Protein samples were buffer exchanged into 200 mM aqueous ammonium acetate (pH 7.2) using a 10 kDa cutoff Amicon 0.5 mL microconcentrators (EMD, Millipore, Billerica, MA). Stock solutions of oligosaccharides (10 mM) were prepared by dissolving a known amount of solid compound in milliQ water and stored at −20 °C until used. Nanoelectrospray (nanoESI) mass spectrometry measurements were performed on a Synapt G2S quadrupole-ion mobility separation-time of flight (Q-IMS-TOF) mass spectrometer (Waters, Manchester, UK) equipped with nanoflow ESI source. NanoESI was performed by applying a voltage of ~1 kV to a platinum wire inserted into the nanoESI tip, which was produced from a borosilicate glass capillary (1.0 mm outside diameter [o.d.], 0.78 mm inside diameter [i.d.]) pulled to ~5 μm o.d. using a P−1000 micropipette puller (Sutter Instruments, Novato, CA). The source temperature was 60 °C and gas flow rates was 2 mL/min. The cone, trap and transfer voltages were 20 V, 3 V, and 1 V, respectively. MassLynx software (version 4.1) was used for data acquisition and processing. Mass spectra were averaged over 300 scans.

Association constants (*K*_a_) for the Agd3^141–364^–oligosaccharide interactions were quantified using the direct ESI-MS assay^79,80^. The binding measurements were carried out at room temperature, the reaction mixtures were prepared by mixing aliquots of the stock solutions to achieve the desired concentration of Agd3^141–364^ (5 μM), oligosaccharide (50–350 μM), and ammonium acetate (200 mM). ESI-MS measurements were performed after 1 h incubation time period. The reported affinities are average values from three replicate measurements performed at a minimum of five different ligand concentrations (protein initial concentration was constant). All mass spectra were corrected, when needed, for the occurrence of nonspecific carbohydrate–protein binding during the ESI process using the reference protein method^81^. *K*_a_ values were determined from the abundance ratio (*R*) of the ligand-bound (*PL*) to free protein (*P*) ions, after correction for nonspecific ligand binding, and the initial concentrations of protein ([*P*]_0_) and ligand ([*L*]_0_), Eq (1):

$$K_a = R/\left( {\left[ L \right]_0-\left( {R/\left[ {1 + R} \right]} \right)[P]_0} \right)$$

where *R* is taken to be equal^79^ to the corresponding concentration ratio ([*PL*]/[*P*]) in solution, Eq (2):

$$R = \frac{{{\sum} {Ab(PL)} }}{{{\sum} {Ab(P)} }} = \frac{{[PL]}}{{[P]}}$$

### Phylogenetics

The amino acid sequence of Agd3 from *A. fumigatus* submitted to BLASTP^82^ with a cutoff of 1e-05 identified 1560 sequences from 314 species. 744 sequences with between 20 and 95% sequence identity to *A. fumigatus* Agd3 and with >50% coverage were downloaded. Agd3 was also submitted to JackHMMER^83^. 121 representative sequences were selected from the BLASTP search and five from JackHMMER, by eliminating those with pairwise identity over 80%, removing protein fragments, allowing for a maximum of one sequence per species. Sequences were aligned by MUSCLE^68^ and MEGA7^84^ then used to construct a maximum-likelihood tree using sites with >95% coverage and 250 bootstrap replicates. Branches were retained at a bootstrap level of 0.7 and the tree was viewed using FigTree (http://tree.bio.ed.ac.uk/software/figtree/).

### Reporting summary

Further information on research design is available in the Nature Research Reporting Summary linked to this article.

## Supplementary information

Supplementary Information

Description of Additional Supplementary Files

Supplementary Data 1

Supplementary Data 2

Reporting Summary

## Data Availability

Crystallographic data that support the findings of this study have been deposited in the Protein Data Bank with the accession codes 6NWZ [10.2210/pdb6NWZ/pdb]. The authors declare that all other data supporting the findings of this study are available within the paper and its supplementary information files. The source data underlying Figs. 4b–d, 5b, 6b–d, 7a–c, and Supplementary Figs. 2 and 5 are provided as a Source Data file.

## Source data

Source Data
