# Supplementary material for: Structural and biochemical characterization of the exopolysaccharide deacetylase Agd3 required for Aspergillus fumigatus biofilm formation
Source: Nat Commun. 2020 May 15;11:2450. doi: 10.1038/s41467-020-16144-5 (PMC7229062; doi:10.1038/s41467-020-16144-5)
Supplement: Supplementary file 1 — Supplementary Information [file 41467_2020_16144_MOESM1_ESM.pdf]

Supplemental information

Aspergillus fumigatus Agd3 is a novel carbohydrate deacetylase involved in biofilm formation

Natalie C. Bamford, François Le Mauff et al.

Supplementary Figures

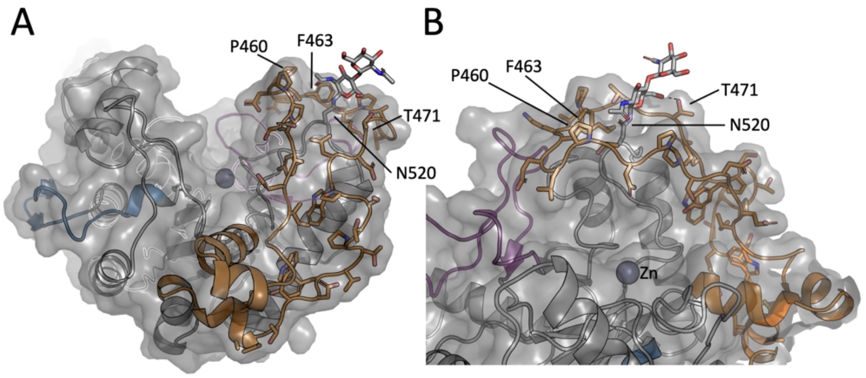

Supplementary Figure 1. The CE domain of Agd3 forms a deep cleft around the metal-binding site. (A) Cartoon representation, with transparent surface, of Agd3 oriented so the CE domain is in the foreground and the (β/α/β)-domain and the C-terminal domain are going into the page. (B) Close up of the CE domain from the side of the (β/α)-barrel of the CE domain. The structural insertions are coloured as in Fig 3A. Residues that interact with the N520 glycan are labeled.

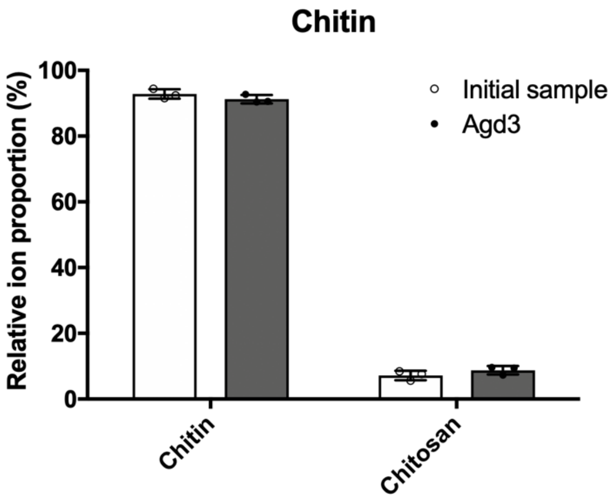

Supplementary Figure 2: Agd3 is unable to deacetylate β-1,4-*N*-acetylglucosamine-containing oligosaccharides. MALDI-TOF MS analysis of chitin and chitosan oligosaccharides incubated for 24 h with 10 µM Agd3. Data points represent relative proportion of fully acetylated (chitin), or partially deacetylated (chitosan), averaged over each oligomer length (trisaccharide to decasaccharide, thus seven MS peaks per condition). The error bars represent SD over three individual experiments.

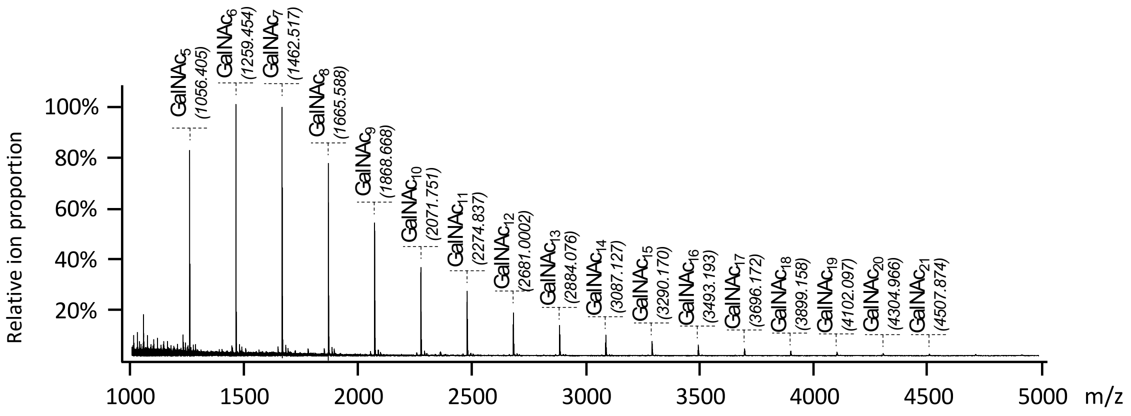

Supplementary Figure 3:_MALDI-TOF MS analysis of GalNAc oligosaccharides purified from *A. fumigatus* biofilm. As previously reported, Sph3-digestion produced GalNAc homopolymers only (1).

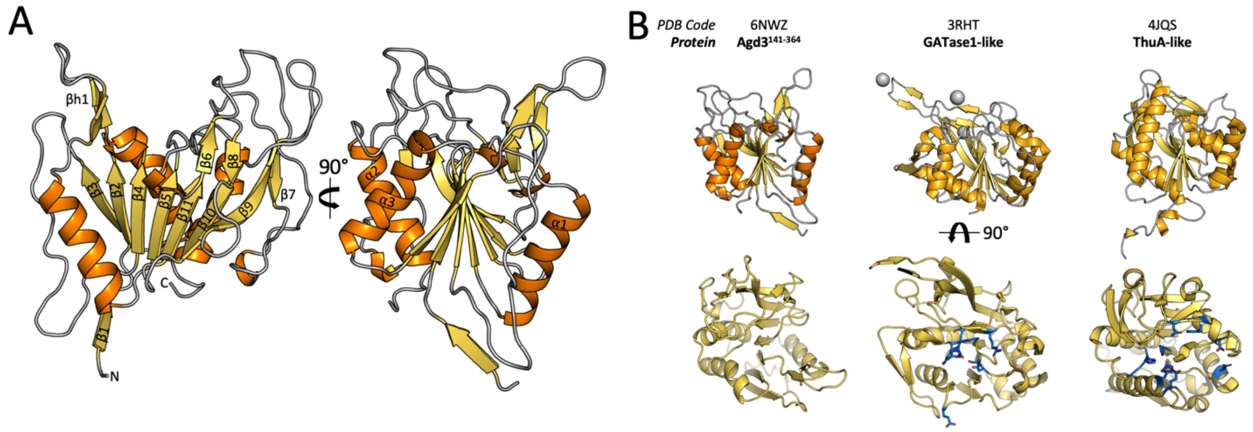

Supplementary Figure 4. Agd3 contains a reductase-like fold. (A) Cartoon representation of the α/β/α-domain of Agd3 with helices in orange and β-strands in yellow. (B) Comparison of the Agd3 α/β/α-domain with GATase1-like (PDB 3RHT) and ThuA-like proteins (PDB 4JQS). The most highly conserved surface exposed residues of both GATase-like and ThuA-like proteins are shown in blue and occur at the C-termini of the central β-sheets.

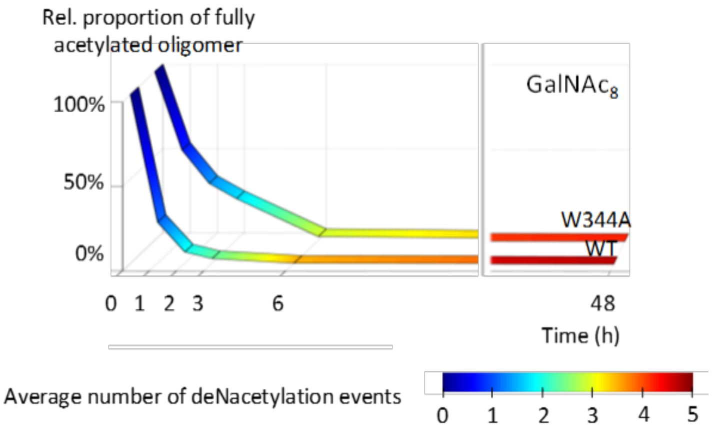

Supplementary Figure 5: Time course of synthetic α-1,4-(GalNAc)$_8$ deacetylation by Agd3^{WT} or Agd3^{W344A}. Products were analyzed by MALDI-TOF MS and expressed as the relative amount of unmodified substrate. The average number of deacetylation events is indicated by the colour legend. Graph represents the average of five replicate experiments.

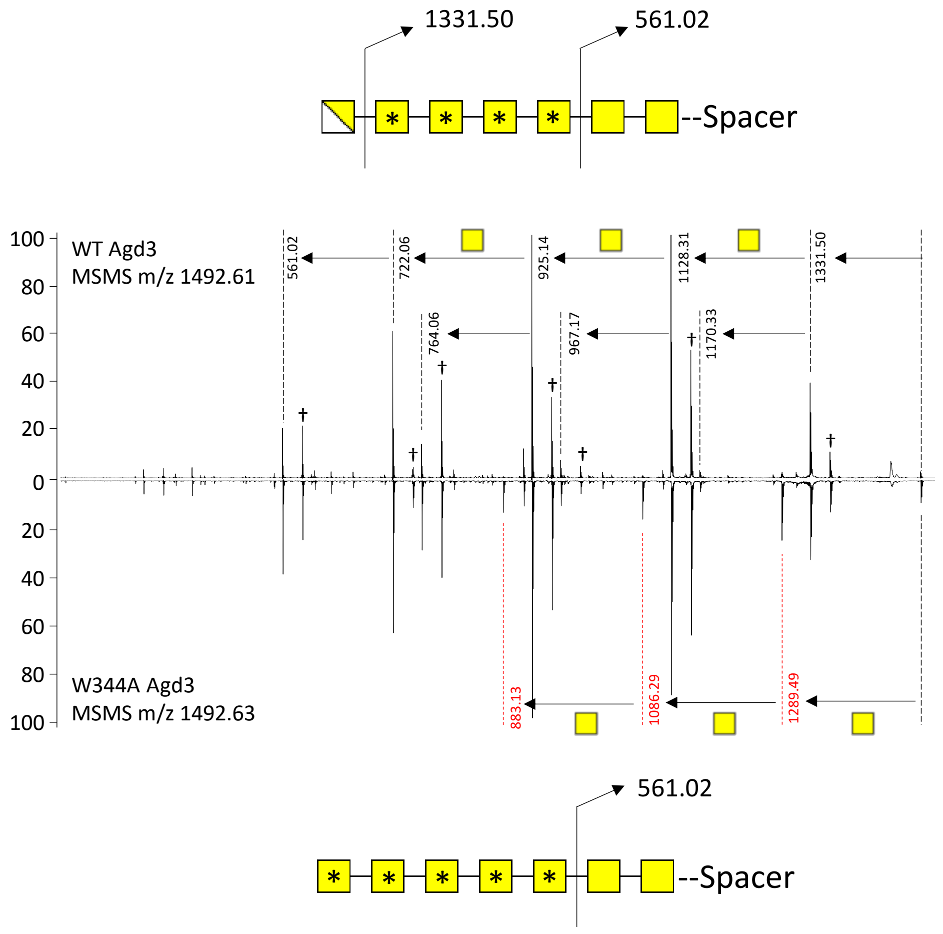

Supplementary Figure 6: Deacetylation of heptasaccharide by Agd3^{WT} (top) and Agd3^{W344A} (bottom) analyzed by MALDI-TOF MS-MS fragmentation. Oligosaccharide diagrams indicates the potential location of the second deacetylation event (shown as a *). Yellow squares represent GalNAc, and half-yellow squares represent GalN units. Synthetic oligosaccharides have a chemical "spacer" at their reducing end. For Agd3^{W344A} the primary deacetylation event can be any of the 5 GalNAc located at the non-reducing end of the oligosaccharide, and is therefore not indicated on the schematic. Y fragmentation ions are reported, ions from B fragmentation are indicated by a †. Ions in red are specific to Agd3^{W344A}, they exist due to the absence of de-*N*-acetylation on the non-reducing termini.

Supplementary Tables

Supplementary Table 1. Summary of data collection and refinement statistics
(Values in parentheses correspond to the highest resolution shell.)

	**Zn-SAD Dataset**	**Agd3**
**Data collection**		
Beamline	NSLS-II 17ID-2	NSLS-II 17ID-2
Wavelength (Å)	1.28308	1.28308
Space group	$P\ 6_1\ 2\ 2$	$P\ 6_1\ 2\ 2$
Unit-cell parameters (Å,°)	$a = b = 78.0$, $c = 640.3$, $\alpha = \beta = 90.0$, $\gamma = 120$	$a = b = 78.0$, $c = 640.3$, $\alpha = \beta = 90.0$, $\gamma = 120$
Resolution (Å)	29.2 – 2.70 (2.79 – 2.70)	29.9 – 2.60 (2.69 – 2.60)*
Total no. of reflections	3,932,103 (122015)	74,628 (7172)
No. of unique reflections	33,646 (3207)	37,314 (487)
Redundancy	116.9 (37.6)	2.0 (14.7)
Completeness (%)	99.6 (98.3)	86.1 (13.6)*
Average $I/\sigma(I)$	29.2 (2.2)	17.9 (1.8)
R_{merge} (%)[1]	19.75 (188)	2.46 (25.7)
$CC_{1/2}$[2]	100 (93.5)	99.9 (96.8)
**Refinement**		
R_{work}[3] / R_{free}[4]		19.0/24.2
No. of atoms		
Protein		5002
Water		120
Zn		6
Average B-factors (Å^2)[5]		47.5
Protein		46.8
Water		41.1
Zn		96.5
RMS deviations		
Bond lengths (Å)		0.003
Bond angles (°)		0.57
Ramachandran plot[6]		
Total favoured (%)		96.4
Total allowed (%)		99.9
Coordinate error (Å)[7]		0.3
PDB code		6NWZ

*Atypical completeness is due to the anisotropic nature of the data. The resolution only extended to 2.6Å in one dimension

[1]$R_{merge} = \sum\sum | I(k) - \langle I \rangle | / \sum I(k)$ where $I(k)$ and $\langle I \rangle$ represent the diffraction intensity values of the individual measurements and the corresponding mean values. The summation is over all unique measurements.

[2]$CC_{1/2}$ is the ratio of Pearson correlation coefficients ($CC = \sum(x-\langle x \rangle)(mL-\langle mL \rangle)/[\sum(x-\langle x \rangle)^2\sum(mL-\langle mL \rangle)^2]^{1/2}$) between random half-sets of data.

[3]$R_{work} = \sum ||F_{obs}| - k|F_{calc}|| / |F_{obs}|$ where F_{obs} and F_{calc} are the observed and calculated structure factors, respectively.

[4]R_{free} is the sum extended over a subset of reflections (5.0%) excluded from all stages of the refinement.

[5]As calculated using MolProbity (2).

[6]Maximum-Likelihood Based Coordinate Error, as determined by PHENIX (3)

Supplementary Table 2. Strains and primers used for Agd3

Primers or Strain	Sequence or Description	Source or reference
**Primers**		
Agd3Pp 141-NdeI	GGGCATATGTCTCACGGTTCCGTTTTGTCCAAC	This study
Agd3Pp 806-XhoI	GGCTCGAGTTACAAAGCGATTGGAGTGGAC	This study
pETHis	GGGAGTCATCGTATGGGCAGCAGCCATCATCATCATC	This study
Agd3Pp 806-KpnI	GGGGGTACCTTACAAAGCGATTGGAGTGGAC	This study
Agd3Pp 362-MlyI	GGGAGTCATCGTGGTTTGTACGCTGGTCACAGAAGAG	This study
Agd3Pp 366-MlyI	GGGAGTCATCGTGGTCACAGAAGAGTTAACTTGAAC	This study
Agd3Pp 733-KpnI	GGGGGTACCTTAACCACACTGATCTCTCTGGTATCTAG	This study
365 EcoRI F	GTTTGTACGCTGAATTCCACAGAAGAG	This study
365 EcoRI R	CTCTTCTGTGGAATTCAGCGTACAAAC	This study
Agd3Pp D377N F	CACTCAGATCAACGACATGTTCTTG	This study
Agd3Pp D377N R	CAAGAACATGTCGTTGATCTGAGTG	This study
Agd3Pp D378N F	CACTCAGATCGACAACATGTTCTTG	This study
Agd3Pp D378N R	CAAGAACATGTTGTCGATCTGAGTG	This study
Agd3Pp H510A F	CACATCTCCGCTACTTTCACTCACG	This study
Agd3Pp H510A R	CGTGAGTGAAAGTAGCGGAGATGTG	This study
Agd3Pp H514A F	CCACACTTTCACTGCTGAGGAACAG	This study
Agd3Pp H514A R	CTGTTCCTCAGCAGTGAAAGTGTGG	This study
Agd3Pp H668A F	GGTTACATGTTCGCTCAGGCTAACTTG	This study
Agd3Pp H668A R	CAAGTTAGCCTGAGCGAACATGTAACC	This study
Agd3Pp R613A F	CAGGTTAACCCAGCTTGGGCTACTAG	This study
Agd3Pp R613A R	CTAGTAGCCCAAGCTGGGTTAACCTG	This study
Agd3Pp M666A F	GACGGTTACGCTTTCCACCAGGCTAAC	This study
Agd3Pp M666A R	GTTAGCCTGGTGGAAAGCGTAACCGTC	This study
Agd3Pp W292A F	CACTGAGGGTTTGGCTCATTACCCAG	This study
Agd3Pp W292A R	CTGGGTAATGAGCCAAACCCTCAGTG	This study
Agd3Pp W344A F	GTTTCGCTACTGACGCTTCCGCTACTTC	This study
Agd3Pp W344A R	GAAGTAGCGGAAGCGTCAGTAGCGAAAC	This study
Agd3Pp D242A F	GAATGGTTCAGTTCGCTGTTTACCCAG	This study
Agd3Pp D242A R	CTGGGTAAACAGCGAACTGAACCATTC	This study
364stop F	CTAGAGGTTTGTACTAGGAATTCGAG	This study
364stop R	CTCGAATTCCTAGTACAAACCTCTAG	This study
**Strains**		
Af293	Wild-type pathogenic strain of *A. fumigatus*	P. Magee
Af293 Δ*agd3*	Wild-type pathogenic strain of *A. fumigatus* with split marker, double homologous recombination to disrupt *agd3*	(4)
PichiaPink Strain 1	*P. pastoris* laboratory expression strain: *ade2*	Invitrogen
PichiaPink Strain 4	*P. pastoris* laboratory expression strain: *ade2, prb1, pep4*	Invitrogen
TOP10	*E. coli* cloning strain	Invitrogen
Origami2 (DE3)	*E. coli* laboratory expression strain: Tet^r Str^r (DE3), *trbX gor*	Stratagene

Supplementary References

1. Le Mauff F, et al. Molecular mechanism of Aspergillus fumigatus biofilm disruption by fungal and bacterial glycoside hydrolases. *J Biol Chem* **294** 13833-13849

2. Chen VB, et al. (2012) MolProbity: all-atom structure validation for macromolecular crystallography. Crystallography of biological macromolecules. (International Union of Crystallography, Chester, England), pp 694–701. 2nd Ed.

3. Adams PD, et al. (2010) PHENIX: a comprehensive Python-based system for macromolecular structure solution. *Acta crystallographica Section D, Biological crystallography* 66(Pt 2):213–221.

4. Lee MJ, et al. (2016) Deacetylation of fungal exopolysaccharide mediates adhesion and biofilm formation. *mBio* 7(2):e00252–16.

5. Gravelat FN, et al. (2010) *Aspergillus fumigatus* MedA governs adherence, host cell interactions and virulence. *Cell Microbiol* 12(4):473–488.

6. Fontaine T, et al. (2011) Galactosaminogalactan, a new immunosuppressive polysaccharide of *Aspergillus fumigatus*. *PLoS Pathog* 7(11):e1002372.

7. Gravelat FN, Askew DS, Sheppard DC (2012) Targeted gene deletion in *Aspergillus fumigatus* using the hygromycin-resistance split-marker approach. *Methods in molecular biology* 845(Chapter 8):119–130.

8. Kelley LA, Sternberg MJE (2009) Protein structure prediction on the Web: a case study using the Phyre server. *Nature protocols* 4(3):363–371.

9. Steentoft C, et al. (2013) Precision mapping of the human O-GalNAc glycoproteome through SimpleCell technology. *The EMBO journal* 32(10):1478–1488.

10. Sievers F, Higgins DG (2018) Clustal Omega for making accurate alignments of many protein sequences. *Protein science: a publication of the Protein Society* 27(1):135–145.

11. Edgar RC (2004) MUSCLE: multiple sequence alignment with high accuracy and high throughput. *Nucleic acids research* 32(5):1792–1797.

12. Sheldrick GM (2008) A short history of SHELX. *Acta Crystallogr, A, Found Crystallogr* 64(Pt 1):112–122.

13. Sheldrick GM (2010) Experimental phasing with SHELXC/ D/ E: combining chain tracing with density modification. *Acta crystallographica Section D, Biological crystallography* 66(4):479–485.

14. Adams PD, et al. (2002) PHENIX: building new software for automated crystallographic structure determination. *Acta crystallographica Section D, Biological crystallography* 58(Pt 11):1948–1954.

15. Emsley P, Cowtan K (2004) Coot: model-building tools for molecular graphics. *Acta crystallographica Section D, Biological crystallography* 60(Pt 12 Pt 1):2126–2132.

16. Morin A, et al. (2013) Collaboration gets the most out of software. *eLife* 2:e01456.

17. Potterton E, Briggs P, Turkenburg M, Dodson E (2003) A graphical user interface to the CCP4 program suite. *Acta crystallographica Section D, Biological crystallography* 59(Pt 7):1131–1137.

18. Winn MD, et al. (2011) Overview of the CCP4 suite and current developments. *Acta crystallographica Section D, Biological crystallography* 67(Pt 4):235–242.

19. Collaborative Computational Project Number 4 (1994) The CCP4 suite: programs for protein crystallography. *Acta crystallographica Section D, Biological crystallography* 50(5):760–763.

20. Strong M, et al. (2006) Toward the structural genomics of complexes: crystal structure of a PE/PPE protein complex from Mycobacterium tuberculosis. *Proc Nat Acad Sci* 103(21):8060–8065.

21. Kitova EN, El-Hawiet A, Schnier PD, Klassen JS (2012) Reliable Determinations of Protein–Ligand Interactions by Direct ESI-MS Measurements. Are We There Yet? *Journal of The American Society for Mass Spectrometry* 23(3):431–441.

22. Wang W, Kitova EN, Klassen JS (2003) Influence of solution and gas phase processes on protein-carbohydrate binding affinities determined by nanoelectrospray Fourier transform ion cyclotron resonance mass spectrometry. *Anal Chem* 75(19):4945–4955.

23. Sun J, Kitova EN, Wang W, Klassen JS (2006) Method for distinguishing specific from nonspecific protein-ligand complexes in nanoelectrospray ionization mass spectrometry. *Anal Chem* 78(9):3010–3018.

24. Potter SC, et al. (2018) HMMER web server: 2018 update. *Nucleic acids research* 46(W1):W200–W204.

25. Kumar S, Stecher G, Tamura K (2016) MEGA7: Molecular Evolutionary Genetics Analysis Version 7.0 for Bigger Datasets. *Molecular biology and evolution* 33(7):1870–1874.
