# Supplementary material for: Structural and biochemical characterization of the exopolysaccharide deacetylase Agd3 required for Aspergillus fumigatus biofilm formation
Source: Nat Commun. 2020 May 15;11:2450. doi: 10.1038/s41467-020-16144-5 (PMC7229062; doi:10.1038/s41467-020-16144-5)
Supplement: Supplementary file 2 — Description of Additional Supplementary Files [file 41467_2020_16144_MOESM2_ESM.pdf]

Description of Additional Supplementary Files

File Name: Supplementary Data 1
Description: Agd3 Blast results

File Name: Supplementary Data 2
Description: Organisms in Tree
