# Supplementary material for: Structural and biochemical characterization of the exopolysaccharide deacetylase Agd3 required for Aspergillus fumigatus biofilm formation
Source: Nat Commun. 2020 May 15;11:2450. doi: 10.1038/s41467-020-16144-5 (PMC7229062; doi:10.1038/s41467-020-16144-5)
Supplement: Supplementary file 5 — Reporting Summary [file 41467_2020_16144_MOESM5_ESM.pdf]

nature research

Corresponding author(s): *Double-blind peer review submissions: write DBPR and your manuscript number here instead of author names.*

Last updated by author(s): *YYYY-MM-DD*

Reporting Summary

Nature Research wishes to improve the reproducibility of the work that we publish. This form provides structure for consistency and transparency in reporting. For further information on Nature Research policies, see Authors & Referees and the Editorial Policy Checklist .

Statistics

For all statistical analyses, confirm that the following items are present in the figure legend, table legend, main text, or Methods section.

n/a	Confirmed	
☐	☒	The exact sample size (*n*) for each experimental group/condition, given as a discrete number and unit of measurement
☐	☒	A statement on whether measurements were taken from distinct samples or whether the same sample was measured repeatedly
☐	☒	The statistical test(s) used AND whether they are one- or two-sided<br>*Only common tests should be described solely by name; describe more complex techniques in the Methods section.*
☒	☐	A description of all covariates tested
☐	☒	A description of any assumptions or corrections, such as tests of normality and adjustment for multiple comparisons
☐	☒	A full description of the statistical parameters including central tendency (e.g. means) or other basic estimates (e.g. regression coefficient) AND variation (e.g. standard deviation) or associated estimates of uncertainty (e.g. confidence intervals)
☐	☒	For null hypothesis testing, the test statistic (e.g. *F*, *t*, *r*) with confidence intervals, effect sizes, degrees of freedom and *P* value noted<br>*Give P values as exact values whenever suitable.*
☒	☐	For Bayesian analysis, information on the choice of priors and Markov chain Monte Carlo settings
☒	☐	For hierarchical and complex designs, identification of the appropriate level for tests and full reporting of outcomes
☒	☐	Estimates of effect sizes (e.g. Cohen's *d*, Pearson's *r*), indicating how they were calculated

Our web collection on statistics for biologists contains articles on many of the points above.

Software and code

Policy information about availability of computer code

Data collection	Data collection at NSLS II used in house software. Bruker Flex Control v.3.4 (build 135), SoftMax Pro v. 5.4.1, Zen 2010 LSM780 v.6.0.0.320, MassLynx software v4.1
Data analysis	Phenix through SBGrid, CCP4 v6.5.020, ShelX suite v2013, FigTree v1.4, Geneious Prime v2019.4, Mega7, Pymol 2.0, Prism7, Bruker Flex Analysis v.3.4 (build 76), Matlab R2018b and Excel Office 365 ProPlus

For manuscripts utilizing custom algorithms or software that are central to the research but not yet described in published literature, software must be made available to editors/reviewers. We strongly encourage code deposition in a community repository (e.g. GitHub). See the Nature Research guidelines for submitting code & software for further information.

Data

Policy information about availability of data

All manuscripts must include a data availability statement. This statement should provide the following information, where applicable:

- Accession codes, unique identifiers, or web links for publicly available datasets
- A list of figures that have associated raw data
- A description of any restrictions on data availability

Crystallographic data that support the findings of this study have been deposited in the Protein Data Bank with the accession codes 6NWZ. The authors declare that all other data supporting the findings of this study are available within the paper and its supplementary information files.

Field-specific reporting

Please select the one below that is the best fit for your research. If you are not sure, read the appropriate sections before making your selection.

Life sciences study design

All studies must disclose on these points even when the disclosure is negative.

Sample size	Statistical methods were not employed to determine sample size. Sample size was chosen to be greater or equal to triplicate when possible, with multiple experiments. These sample sizes were sufficient as they are consistent with general practice for biochemical and protein work, or previously published studies.
Data exclusions	Data was only excluded due to human error. This is only for the ELISA based assays in which blocking or washing was not effective and the negative control appeared similar in value to the positive control.
Replication	In our hands results were reproducible as shown by the data. Each experiment was run more than once as stated in the legend for each experiment. Some issues occurred when using different preparations of soluble GAG from Aspergillus cultures as each preparation seemed to have differing concentrations of polysaccharide. This was mitigated by using one preparation for all experiments preformed for each assay to remove this as a variable. In the case of the hyphal staining and imaging, this experiment was performed by two different researchers with similar results.
Randomization	This is not applicable. There were no participants or samples that could be randomly assigned to groups or treatments. This study was in vitro experiments only.
Blinding	This is not applicable. These experiments were in vitro and thus not blinded.

Reporting for specific materials, systems and methods

We require information from authors about some types of materials, experimental systems and methods used in many studies. Here, indicate whether each material, system or method listed is relevant to your study. If you are not sure if a list item applies to your research, read the appropriate section before selecting a response.

Materials & experimental systems

n/a	Involved in the study	
☐	☒	Antibodies
☒	☐	Eukaryotic cell lines
☒	☐	Palaeontology
☒	☐	Animals and other organisms
☒	☐	Human research participants
☒	☐	Clinical data

Methods

n/a	Involved in the study	
☒	☐	ChIP-seq
☒	☐	Flow cytometry
☒	☐	MRI-based neuroimaging

Antibodies

Antibodies used	Polyclonal Donkey–anti-mouse secondary antibody conjugated to horseradish peroxidase (Cedarlane, Lot 41-112-020317), Monoclonal mouse-anti-His-Tag (Abgent, clone 6AT18).
Validation	The primary anti-His-Tag antibody was clone number 6AT18 from Abgent and was used at the company advised concentration. According to Abgent "Purified recombinant HIS-tagged fusion protein and poly-HIS peptide were used to produced this monoclonal antibody. This antibody is purified through a protein G column, followed by dialysis against PBS." This antibody has been used in an ELISA based experiment in a peer reviewed publication: Malmirchegini, G. R., Sjodt, M., Shnitkind, S., Sawaya, M. R., Rosinski, J., Newton, S. M., Klebba, P. E., and Clubb, R. T. (2014) Novel mechanism of hemin capture by Hbp2, the hemoglobin-binding hemophore from Listeria monocytogenes. J Biol Chem. 289, 34886–34899
